# Iloprost as an acute kidney injury-triggering agent in severely atherosclerotic patients

**DOI:** 10.5830/CVJA-2015-051

**Published:** 2016

**Authors:** Mehtap Erkmen Uyar, Piril Yucel, Sena Ilin, Zeynep Bal, Saliha Yildirim, Ahmet Senol Uyar, Tankut Akay, Emre Tutal, Siren Sezer

**Affiliations:** Department of Internal Medicine, Baskent University, Ankara, Turkey; Department of Internal Medicine, Baskent University, Ankara, Turkey; Department of Internal Medicine, Baskent University, Ankara, Turkey; Department of Internal Medicine, Baskent University, Ankara, Turkey; Department of Internal Medicine, Baskent University, Ankara, Turkey; Department of Anesthesiology, Ulucanlar Eye Education and Research Hospital, Ankara, Turkey; Department of Cardiovascular Surgery, Baskent University, Ankara, Turkey; Department of Nephrology, Baskent University, Ankara, Turkey; Department of Nephrology, Baskent University, Ankara, Turkey

**Keywords:** iloprost, acute kidney injury, severe atherosclerosis

## Abstract

**Background:**

Iloprost, a stable prostacyclin analog, is used as a rescue therapy for severe peripheral arterial disease (PAD). It has systemic vasodilatory and anti-aggregant effects, with severe vasodilatation potentially causing organ ischaemia when severe atherosclerosis is the underlying cause. In this study, we retrospectively analysed renal outcomes after iloprost infusion therapy in 86 patients.

**Methods:**

Eighty-six patients with PAD who received iloprost infusion therapy were retrospectively analysed. Clinical and biochemical parameters were recorded before (initial, Cr1), during (third day, Cr2), and after (14th day following the termination of infusion therapy, Cr3) treatment. Acute kidney injury (AKI) was defined according to KDIGO guidelines as a ≥ 0.3 mg/dl (26.52 μmol/l) increase in creatinine levels from baseline within 48 hours.

**Results:**

Cr2 (1.46 ± 0.1 mg/dl) (129.06 ± 8.84 μmol/l) and Cr3 (1.53 ± 0.12 mg/dl) (135.25 ± 10.61 μmol/l) creatinine levels were significantly higher compared to the initial value (1.15 ± 0.6 mg/dl) (101.66 ± 53.04 μmol/l). AKI was observed in 36 patients (41.86%) on the third day of iloprost infusion. Logistic regression analysis revealed smoking and not using acetylsalicylic acid as primary predictors (*p* = 0.02 and *p* = 0.008, respectively) of AKI during iloprost treatment. On the third infusion day, patients’ urinary output significantly increased (1813.30 ± 1123.46 vs 1545.17 ± 873.00 cm^3^) and diastolic blood pressure significantly decreased (70.07 ± 15.50 vs 74.14 ± 9.42 mmHg) from their initial values.

**Conclusion:**

While iloprost treatment is effective in patients with PAD who are not suitable for surgery, severe systemic vasodilatation can cause renal ischaemia, resulting in nonoliguric AKI. Smoking, no acetylsalicylic acid use, and lower diastolic blood pressure are the clinical risk factors for AKI during iloprost treatment.

## Background

Iloprost is a stable epoprostenol (prostacyclin, PGI2) analog that mimics the effects of prostacyclin in the microvascular blood flow, namely, inhibition of platelet aggregation, leukocyte–vessel interaction and vasodilatation.^1^,^2^ A superior chemical stability confers iloprost with a longer half-life than that of prostacyclin, giving it an advantage as a therapeutic agent for the treatment of many cardiovascular and pulmonary diseases.^3^ Iloprost also inhibits platelet aggregation and leukocyte activation, and leads to vasodilatation in ischaemic tissue after ischaemia/reperfusion (I/R) injury.^4^,^5^ Iloprost is used as a rescue therapy for patients with severe obstructive peripheral arterial disease (PAD) who cannot tolerate surgery.

Renoprotective effects of iloprost have been reported in contrast-induced nephropathy and I/R injury.^6^ In these cases, iloprost infusion was administered either at a low dose or for a short duration, causing vasodilation without systemic hypotension.^7^,^8^ On the other hand, during rescue therapy for severe atherosclerosis, its use may lower blood pressure,^9^ leading to tissue ischaemia, renal hypoperfusion and acute kidney injury.^10^

Because of iloprost’s hypotension-inducing potent systemic vasodilatory effect, several potential safety issues should be considered in these patients.^11^ This ischaemia-triggering hypoperfusion effect of iloprost may cause organ dysfunction, such as acute kidney injury, especially in patients with co-morbidities. On the basis of the systemic hypotensive effect of iloprost and our clinical experience of patients with acute kidney injury under iloprost treatment, we retrospectively analysed the effects of iloprost infusion therapy on renal outcomes in 86 patients.

## Methods

We retrospectively analysed patients with severe PAD who received iloprost infusion therapy at a dose of 1 ng/kg/min between January 2011 and January 2012 at Baskent University Hospital, Ankara, Turkey. Severe PAD was detected with non-invasive tests, including ankle–brachial index < 0.40 and absent blood flow on duplex ultasonography. Among these, 86 patients were selected according to the following exclusion criteria: (1) malignant disease, (2) rheumatological or chronic inflammatory disease of unknown origin, (3) history of systemic vasculitis, (4) unstable heart failure (ejection fraction < 50%) during infusion therapy, (5) chronic liver failure, (6) systemic infective or non-infective inflammatory diseases, (7) evidence of end-stage renal disease (ESRD), (8) younger than 18 years of age. An informed consent was obtained from all subjects of the study.

According to the treatment protocol, patients received iloprost infusion at a dose of 1 ng/kg/min for 10–14 days. Iloprost in a 100-ml isotonic solution was infused during a six-hour period via the intravenous route. Nausea, flushing, headache and thrombophlebitis were recorded as drug-related side effects. In accordance with the Kidney Disease Improving Global Outcomes (KDIGO) guidelines, acute kidney injury (AKI) was defined as ≥ 0.3 mg/dl (26.52 μmol/l) increase in creatinine levels from baseline within 48 hours.^12^

The following parameters were collected retrospectively from clinical charts: (1) age; (2) gender; (3) smoking status; (4) presence of diabetes, hypertension, dyslipidaemia [serum highdensity lipoprotein cholesterol (HDL-C) < 40 mg/dl (1.04 mmol/l) and/or low-density lipoprotein cholesterol (LDL-C) > 130 mg/dl (3.37 mmol/l) and/or triglycerides > 300 mg/dl (3.39 mmol/l)], or ischaemic heart disease; (4) urinary output; (5) use of statins, acetylsalicylic acid (ASA), clopidogrel, low-molecularweight heparin (LMWH), angiotensin converting enzyme inhibitors (ACEI), or angiotensin receptor blockers (ARB); (6) daily systolic, diastolic and mean arterial (2 × diastolic pressure + systolic pressure)/3) blood pressure measurement data; (7) haemoglobin, sodium, potassium, calcium, phosphorus, albumin, blood urea nitrogen (BUN), creatinine, and estimated glomerular filtration rate (eGFR) (MDRD equation) values.

Clinical and biochemical parameters were collected before (baseline), during (third day of infusion therapy) and after (two weeks after cessation of infusion therapy; 28th day) iloprost treatment, and mean values were determined as arithmetic means. Baseline values were defined as those measured at admission to the in-patient clinic. Office blood pressure levels were recorded.

## Statistical analysis

Statistical analyses were performed using SPSS software (Statistical Package for the Social Sciences, version 15.0, SPSS Inc, Chicago, IL, USA). Subjects were grouped according to the absence of AKI, as the normal renal function group (*n* = 50), and presence of AKI, as the AKI group (*n* = 36).

Normality of data was analysed using the Kolmogorov–Smirnov test. All numerical variables with normal distributions were expressed as means ± standard deviations (SD), while variables with skewed distributions were expressed as medians and interquartile ranges (IR). Categorical variables were expressed as percentages and compared using the chi-squared test. Normally distributed numerical variables were analysed by the independent samples *t*-test, one-way ANOVA (*post-hoc* Tukey), or paired samples *t*-test. Numerical variables with a skewed distribution were compared using the Mann–Whitney *U*- and Kruskal–Wallis tests. Spearman and Pearson correlation tests were used for correlation analyses. A binary logistic regression analysis was performed to assess the major determinant of AKI between correlated variables. A Kaplan–Meier survival analysis was used to compare 30-day patient survival between the two groups. A *p*-value < 0.05 was considered statistically significant.

## Results

The clinical features of the patients are summarised in Table 1. According to KDIGO criteria, 36 (41.86%) patients were diagnosed with AKI on the third day of iloprost infusion therapy. Co-morbidities and drug use (ASA, clopidogrel, LMWH, statin, ACEI) rates were similar in those with and without AKI (Table 1).

**Table 1:** Clinical features of the patients

	*Whole study group (n=86)*	*Patients with AKI (n=36)*	*Patients without AKI (n=50)*	*p-value*
Age (years)	65.82 ± 16.7	69.77 ± 12.9	64.24 ± 17.0	0.109
Male gender, n (%)	56 (66.2)	21 (58.3)	35 (70)	0.186
Diabetes mellitus, n (%)	48 (55.8)	22 (61.1)	26 (52)	0.221
Hypertension, n (%)	84 (97.6)	36 (100)	48 (96)	0.196
Ischaemic heart disease, n (%)	40 (46.5)	17 (47.2)	23 (46)	0.542
Dyslipidaemia, n (%)	24 (27.9)	10 (27.8)	14 (28)	0.589
Smoking habbit, n (%)	43 (50)	14 (38.9)	29 (58)	0.063
ASA, n (%)	57 (66.2)	20 (55.6)	37 (74)	0.045
Clopidogrel, n (%)	26 (30.2)	12 (33.3)	14 (28)	0.340
LMWH, n (%)	43 (50)	16 (44.4)	27 (54)	0.265
Statin, n (%)	18 (20.9)	8 (22.2)	10 (20)	0.354
ACEI, n (%)	32 (37.2)	14 (38.9)	18 (36)	0.469
ARB, n (%)	5 (5.8)	2 (5.5)	3 (6)	0.657
Mortality rate at 30 days’ follow up, n (%)	9 (10.4)	8 (22.2)	1 (2)	0.003

ASA, acetylsalicylic acid; LMWH, low-molecular-weight heparin; ACEI, angiotensin converting enzyme inhibitors; ARB, angiotensin receptor blocker.

When the entire study group was analysed, serum creatinine levels recorded on the third and 28th day of treatment (1.53 ± 0.12 and 1.46 ± 0.1 mg/dl, respectively) (135.25 ±10.61 and 129.06 ± 8.84 μmol/l) were significantly higher than the baseline level [1.15 ± 0.6 mg/dl (101.66 ± 53.04 μmol/l), *p* = 0.001 for both]. The BUN level recorded on the third day (30.0 ± 20.7 mg/dl) was significantly higher than the baseline level (23.6 ± 13.7 mg/dl, *p* = 0.014), as was the serum C-reactive protein (CRP) level (81.12 ± 4.67 vs 52.48 ± 4.85 mg/dl, *p* = 0.009). On the third day of the infusion, urinary output was significantly increased from the baseline value (1 813.30 ± 1 123.46 vs 1 545.17 ± 873.0 cm^3^, *p* = 0.012) while eGFR values were significantly lower compared to baseline levels (71.16 ± 43.43 vs 76.98 ± 35.57 ml/min/1.73 m^2^, *p* = 0.01) (Table 2). All patients had a significant decrease from baseline in diastolic blood pressure on the third day of infusion therapy (70.29 ± 14.94 vs 74.37 ± 9.09 mmHg, *p* = 0.011). Patients’ mean arterial pressures were significantly decreased on the third day of therapy (90.57 ± 10.5 vs 86.25 ± 17.9 mmHg, *p* = 0.024).

**Table 2:** The laboratory parameters of the whole study group)

*Laboratory parameters*	*Initial*	*Third day of infusion*	*Two weeks after infusion*	*p-value*
Glucose (mg/dl)	143.9 ± 69.7	135.65 ± 49.39	141.55 ± 66.2	0.062*
(mmol/l)	(7.99 ± 3.87)	(7.53 ± 2.74)	(7.86 ± 3.67)	0.289^§^
BUN (mg/dl)	23.6 ± 13.7	30.0 ± 20.7	24.9 ± 13.5	0.014*
				0.458^§^
Creatinine (mg/dl)	1.15 ± 0.60	1.53 ± 0.12	1.46 ± 0.10	0.001*
(μmol/l)	(101.66 ± 53.04)	(135.25 ± 10.61)	(129.06 ± 8.84)	0.001^§^
Haemoglobin (g/dl)	12.7 ± 2.1	11.8 ± 1.9	11.7 ± 1.6	0.236*^§^
Sodium (mmol/l)	134.9 ± 14.8	137.4 ± 4.9	137.1 ± 15.7	0.228*
				0.117^§^
Potassium (mmol/l)	4.76 ± 0.78	4.11 ± 0.68	4.42 ± 0.92	0.406*
				0.606^§^
Phosphorus	3.45 ± 0.78	3.80 ± 2.18	3.87 ± 1.38	0.865*
(mg/dl)				0.185^§^
Calcium (mg/dl)	8.9 ± 0.6	8.8 ± 0.8	8.9 ± 1.4	0.307*
				0.587^§^
Albumin (g/dl)	3.6 ± 0.7	3.4 ± 0.8	3.2 ± 0.7	0.339*
				0.047^§^
CRP (mg/dl)	52.48 ± 4.85	81.12 ± 4.67	67.05 ± 5.15	0.009*
				0.125^§^
Urinary output	1545.17 ± 873.00	1813.30 ± 1123.46	1447.32 ± 934.63	0.012*
(cm^3^/24 h)				0.406^§^
eGFR (MDRD)	76.98 ± 35.57	71.16 ± 43.43	72.84 ± 53.39	0.01*
(ml/min/1.73 m^2^)				0.04^§^
Systolic blood pressure (mmHg)	122.97 ± 16.51	118.16 ± 26.41	121.71 ± 19.72	0.606*
				0.117^§^
Diastolic blood pressure (mmHg)	74.37 ± 9.09	70.29 ± 14.94	71.20 ± 12.65	0.011*
				0.025§
Mean arterial pressure (mmHg)	90.57 ± 10.5	86.25 ± 17.9	88.04 ± 14.3	0.024*
				0.112

*p-value for initial vs at the third day of infusion; ^§^p-value for initial vs two weeks after infusion.

BUN, blood urea nitrogen; CRP, C-reactive protein; eGFR, estimated glomerular filtration rate.

On the 28th day, eGFR values were significantly lower than baseline values (72.84 ± 53.39 vs 76.98 ± 35.57 ml/min/1.73 m^2^, *p* = 0.01) (Table 2). All patients had a significant decrease in diastolic blood pressure on the 28th day compared to baseline values (71.20 ± 12.65 vs 74.37 ± 9.09 mmHg, *p* = 0.025). A non-significant trend towards a lower blood pressure on the third and 28th days was observed (*p* > 0.05) (Table 2). According to data from the 28th day, renal function improved as BUN levels decreased to baseline values, while the creatinine level was high and eGFR was significantly lower (Table 2).

Patients who developed AKI had significantly higher serum creatinine (*p* = 0.032) and CRP (*p* = 0.012) levels and significantly lower eGFR values (*p* = 0.05) at baseline compared to patients without AKI (Table 3). Those who developed AKI had significantly higher serum BUN (*p* = 0.001) and creatinine (*p* = 0.001) levels and lower eGFR (*p* = 0.001) and systolic (*p* = 0.015), diastolic (*p* = 0.014) and mean arterial (*p* = 0.039) blood pressure values on the third day of infusion compared to patients without AKI (Table 3).

**Table 3:** Laboratory parameters of the whole group

	*Initial*			*Third day of infusion*			*Two weeks after infusion*			
*Laboratory parameters*	*Patients with AKI (n = 36)*	*Patients without AKI (n = 50)*	*p-value*	*Patients with AKI (n = 36)*	*Patients without AKI (n = 50)*	*p-value*	*Patients with AKI (n = 36)*	*Patients without AKI (n = 50*	*p-value*	*p-values within the AKI group*
Glucose (mg/dl)	146.32 ± 67.9	141.21 ± 72.3	> 0.05	120.46 ± 41.7	146.20 ± 47.3	0.037	133.17 ± 61.3	147.10 ± 72.0	> 0.05	0.042*
(mmol/l)	(8.12 ± 3.77)	(7.84 ± 4.01)		(6.69 ± 2.31)	(8.11 ± 2.63)		(7.39 ± 3.40)	(8.16 ± 4.00)		> 0.05^§†^
BUN (mg/dl)	26.0 ± 12.1	22.4 ± 14.7	> 0.05	44.88 ± 21.6	19.40 ± 8.28	0.001	33.08 ± 15.5	18.4 ± 9.1	0.001	0.012*
										0.001^§†^
Creatinine (mg/dl)	1.28 ± 0.60	1.05 ± 0.5	0.032	2.37 ± 1.26	0.94 ± 0.3	0.001	2.14 ± 1.11	0.87 ± 0.3	0.001	0.001*
(μmol/l)	(113.15 ± 53.04)	(92.82 ± 44.20)		(209.51 ± 111.38)	(83.10 ± 26.52)		(189.18 ± 98.12)	(76.91 ± 26.52)		> 0.05^§^
										0.001^†^
Haemoglobin (g/dl)	12.09 ± 2.1	13.04 ± 2.0	> 0.05	11.33 ± 1.8	12.06 ± 1.8	> 0.05	11.88 ± 1.1	12.30 ± 1.6	> 0.05	> 0.05^*§†^
Sodium (mmol/l)	136.91 ± 4.0	133.74 ± 19.1	> 0.05	138.05 ± 6.2	136.91 ± 3.4	> 0.05	139.16 ± 5.6	135.20 ± 20.3	> 0.05	> 0.05^*§†^
Potassium (mmol/l)	4.34 ± 0.5	4.19 ± 0.9	> 0.05	4.17 ± 0.6	4.07 ± 0.7	> 0.05	4.16 ± 0.8	4.17 ± 0.2	> 0.05	> 0.05^*§†^
Phosphorus (mg/dl)	3.72 ± 0.8	3.22 ± 0.6	> 0.05	4.28 ± 2.9	3.28 ± 0.5	> 0.05	4.60 ± 1.6	3.23 ± 0.7	> 0.05	> 0.05^*§†^
Calcium (mg/dl)	8.84 ± 0.5	8.93 ± 0.7	> 0.05	8.98 ± 1.09	8.67 ± 0.6	> 0.05	9.21 ± 1.9	8.78 ± 0.7	> 0.05	> 0.05^*§†^
Albumin (g/dl)	3.53 ± 0.6	3.73 ± 0.8	> 0.05	2.62 ± 1.0	3.42 ± 0.6	> 0.05	2.91 ± 0.7	3.62 ± 0.6	0.001	> 0.05^*§†^
CRP (mg/dl)	67.62 ± 7.7	40.38 ± 4.3	0.012	102.85 ± 9.7	70.67 ± 6.6	0.012	90.62 ± 8.7	52.80 ± 9.3	> 0.05	0.001*
										> 0.05^§^
										0.01^†^
Urinary output	1242.91 ± 990.15	1663.37 ± 736.3	> 0.05	1503.20 ± 1267.09	1918.18 ± 827.1	0.004	1259.63 ± 986.0	1632.96 ± 648.2	> 0.05	0.005*^§^
(cm^3^/24 h)										> 0.05^†^
eGFR (MDRD)	70.22 ± 41.7	84.83 ± 34.3	0.05	36.04 ± 23.4	93.10 ± 40.2	0.001	40.68 ± 25.9	101.03 ± 52.84	0.001	0.0001*^†^
										> 0.05^§^
Systolic blood pressure (mmHg)	121.6 ± 19.5	123.78 ± 14.5	> 0.05	106.8 ± 31.7	125.0 ± 10.8	0.015	116.2 ± 28.6	124.92 ± 20.1	> 0.05	0.002*
										> 0.05^§^
										0.023^†^
Diastolic blood pressure (mmHg)	71.8 ± 10.5	75.90 ± 7.8	> 0.05	61.2 ± 17.5	74.64 ± 8.2	0.014	65.4 ± 16.4	75.71 ± 9.9	0.001	0.023*
										> 0.05^§^
										0.002^†^
Mean arterial pressure (mmHg)	88.40 ± 12.5	91.86 ± 8.9	> 0.05	76.0 ± 21.6	91.42 ± 8.0	0.039	82.36 ± 19.9	92.11 ± 12.1	0.002	0.003*
										> 0.05^§^
										0.04^†^

*p-value for initial vs on the third day of infusion; ^§^p-value for initial vs two weeks after infusion; ^†^p-value for third day vs two weeks after infusion. BUN, blood urea nitrogen; CRP, C-reactive protein; eGFR, estimated glomerular filtration rate.

Serum glucose levels of patients with AKI were significantly higher both compared to their own baseline value (*p* = 0.042) and to the value of the third day of patients without AKI (*p* = 0.037) (Table 3). Patients who developed AKI had significantly higher serum creatinine (*p* = 0.001), BUN (*p* = 0.012), CRP (*p* = 0.001) and urinary output (*p* = 0.005) levels on the third day of infusion compared to baseline values. Among patients who developed AKI, systolic (*p* = 0.002), diastolic (*p* = 0.023) and mean arterial pressures (*p* = 0.003) as well as eGFR (*p* = 0.0001) values were significantly lower on the third day of infusion compared to the baseline value (Table 3).

Drug-related side effects were similar in both patient groups (Table 4). A binary logistic regression analysis of co-morbidities and drugs revealed that smoking, diastolic hypotension, and no ASA use were significant independent predictors (*p* = 0.02, *p* = 0.003 and *p* = 0.008, respectively) for the development of AKI during iloprost treatment.

**Table 4:** Drug-related side effects

*Side effects*	*Patients with AKI (n = 36)*	*Patients without AKI (n = 50)*	*p-value*
Nausea, n (%)	9 (25)	3 (6)	0.172
Flushing, n (%)	1 (2.7)	1 (2)	0.660
Headache, n (%)	1 (2.7)	3 (6)	0.448
Thrombophylebitis, n (%)	0	0	NA

**Fig. 1.**
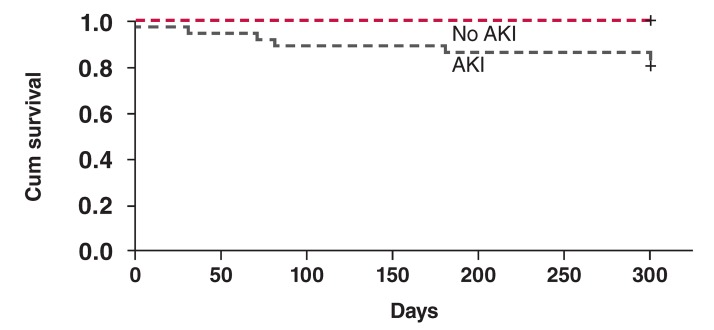
The Kaplan–Meier survival analysis between patients with AKI and those without AKI (at 30-day follow up: 22.2 vs 2%, *p* = 0.001).

We also evaluated factors associated with 30-day mortality and compared survival ratios between the patient groups. The Cox regression analysis revealed that diabetes mellitus (*p* = 0.005) and AKI (*p* = 0.012) are significant determinants of mortality in patients undergoing iloprost infusion therapy. The Kaplan– Meier analysis revealed a significant difference in survival between patients with AKI and those without AKI (at 30-day follow up: 22.2 vs 2%, *p* = 0.001) (Fig. 1).

## Discussion

AKI refers to a rapid and reversible decrease in kidney function that develops within a period of hours or days. In this retrospective study, we assessed the relationship between laboratory and clinical parameters and subsequent changes in kidney function in patients with PAD who developed AKI after iloprost infusion therapy.

We observed that iloprost infusion therapy led to hypotension (systolic, diastolic and mean arterial pressure) and a significant decline in eGFR. Patients who developed AKI were more likely to have worse renal function at the initiation of therapy than other patients. In the multivariate analysis, diastolic hypotension, smoking and lack of ASA treatment were independently associated with an increased risk of developing AKI. In addition, AKI was associated with a higher mortality rate at the 30-day follow up.

In the kidney, prostaglandins uphold the balance between vasodilation and vasoconstriction to maintain homeostasis and physiological kidney function.^13^,^14^ Vasodilator prostaglandins have clinically important side effects that underscore their potential efficacy in the treatment of severe PAD.^15^

In experimental animal studies, iloprost preserved kidney function against anoxia in rabbits,^16^ and had beneficial effects in I/R-induced renal injury in a rat model.^17^ Furthermore, in a clinical study by Spargias *et al.*, iloprost was successfully used to prevent contrast-mediated nephropathy.^9^ However, in these studies that reported iloprost to be a renoprotective agent, the selected doses were as low as 1–2 ng/kg/min and the infusion period lasted approximately four to six hours to avoid systemic hypotension, and dosing was not repeated.^9^,^17^-^19^

Hypotension is the principal, dose-dependent side effect of iloprost. There is evidence that such hypotension is a risk factor for the development of AKI and it is a commonly encountered problem in elderly patients with AKI,^20^-^22^ patients with pre-existing renal insufficiency,^23^,^24^ and patients with low cardiac output states such as myocardial infarction and congestive cardiac failure.^24^-^26^

We observed that patients who received iloprost had a significant decrease in systolic, diastolic and mean arterial pressure compared to baseline, and that relative diastolic hypotension was a significant risk factor for the development of AKI. In their study, Liu *et al.* showed an independent association between the relative decrease in systolic blood pressure and the development of AKI.^27^ Sutton *et al.*^28^ used an ischaemic rat model to demonstrate that the ‘initiation’ phase of AKI, during which renal blood flow is reduced, is the primary determinant of GFR.^6^ Similarly to these studies, our AKI patients had significantly lower diastolic blood pressure, causing decreased renal blood flow and leading to a decline in GFR.

In patients with chronic kidney disease (CKD), the risk of developing AKI is significantly increased.^29^ Co-morbidities such as diabetes, hypertension and proteinuria in hospitalised patients were independently associated with an increased risk of AKI, requiring dialysis.^29^,^30^ Our patients with AKI showed significantly reduced renal function with significantly higher serum creatinine levels and lower eGFR at the initiation of iloprost treatment. These patients were more prone to develop AKI because of the kidney’s sensitivity to disrupted microperfusion or hypotensive ischaemia.

Consistent with these findings, smoking and the lack of ASA use were significant independent predictors for the development of AKI in our patients. Smoking is a major preventable risk factor for atherosclerosis. Exposure to cigarette smoke activates a number of mechanisms predisposing to atherosclerosis, including thrombosis, vascular inflammation, abnormal vascular growth and angiogenesis.^31^-^33^

ASA, the fundamental therapy given for PAD, reduces the risk of cardiovascular events and arterial occlusion. The use of ASA for primary and secondary prevention of cardiovascular events in most patients with PAD is supported by excellent clinical evidence.^34^ Based on these data, we can speculate that the presence of smoking and absence of ASA use were associated with microvascular ischaemia, which made these patients more prone to hypotensive AKI.

The mortality rate in AKI patients with CKD was 3.3 times higher than that of patients without CKD.^35^ In our study, patients with AKI had significantly higher mortality rates over 30 days of follow up. In addition, according to our findings, diabetes mellitus and AKI were significant determinants of mortality in patients undergoing iloprost infusion therapy.

This study has several limitations, many of which are inherent in its retrospective design, including the possibility of missing risk factors that could contribute to a confounding bias. In addition, renal imaging studies were unavailable for all patients. However, the association between iloprost-induced hypotension and AKI was independent and clear. Our results clearly illustrate that relative hypotension may play a key role in the development of AKI during iloprost infusion therapy in patients with altered renal function.

## Conclusion

We conducted a retrospective study to evaluate the risk factors for AKI development and mortality in patients with severe PAD treated with iloprost. We found that patients who developed AKI were more likely to have relative decreases in systolic, diastolic and mean arterial pressures and worse baseline renal function than unaffected patients. We suggest that patients with a smoking habit and those not using ASA are at an increased risk for AKI. In this group of patients we advise iloprost dose reduction and close follow up for evidence of AKI, and discontinuation of iloprost in patients with severe hypotension. As patients with AKI have a higher mortality risk, we suggest that iloprost treatment should be given to selected patients. From our findings, we advise that iloprost should be avoided as it is very likely to cause AKI in patients with CKD or low blood pressure. In addition, we recommend iloprost dose reduction or possible discontinuation for patients receiving iloprost who show evidence of AKI or hypotension. Ultimately, prospective, randomised studies will be needed to address the effects of iloprost infusion therapy on renal outcomes in patients with severe PAD.

## References

[1] Sahsivar MO, Narin C, Kiyici A, Toy H, Ege E, Sarigül A (2009). The effect of iloprost on renal dysfunction after renal I/R using cystatin C and beta2-microglobulin monitoring.. Shock.

[2] Grant SM, Goa KL (1992). Iloprost: a review of its pharmacodynamic and pharmacokinetic properties, and therapeutic potential in peripheral vascular disease, myocardial ischaemia and extracorporeal circulation procedures.. Drugs.

[3] Fink AN, Frishman WH, Azizad M, Agarwal Y (1999). Use of prostacyclin and its analogues in the treatment of cardiovascular disease.. Heart Dis.

[4] Kiris I, Tekin I, Yilmaz N, Sutcu R, Karahan N, Ocal A (2009). Iloprost down-regulates expression of adhesion molecules and reduces renal injury induced by abdominal aortic ischemia-reperfusion.. Ann Vasc Surg.

[5] Ozcan AV, Sacar M, Aybek H (2007). The effects of iloprost and vitamin C on kidney as a remote organ after ischemia/reperfusion of lower extremities.. J Surg Res.

[6] Tumlin JA (2009). Impaired blood flow in acute kidney injury: pathophysiology and potential efficacy of intrarenal vasodilator therapy.. Curr Opin Crit Care.

[7] McCullough PA, Tumlin JA (2009). Prostaglandin-based renal protection against contrast-induced acute kidney injury.. Circulation.

[8] Sketch MH Jr, Whelton A, Schollmayer E (2001). Prevention of contrast media-induced renal dysfunction with prostaglandin E1: a randomized, double-blind, placebo-controlled study.. Am J Ther.

[9] Spargias K, Adreanides E, Giamouzis G (2006). Iloprost for prevention of contrast-mediated nephropathy in high-risk patients undergoing a coronary procedure. Results of a randomized pilot study. Prevention of contrast media-induced renal dysfunction with prostaglandin E1: a randomized, double-blind, placebo-controlled study.. Eur J Clin Pharmacol.

[10] Abuelo JG (2007). Normotensive ischemic acute renal failure.. N Engl J Med.

[11] Spargias K, Adreanides E, Demerouti E (2009). Iloprost prevents contrast-induced nephropathy in patients with renal dysfunction undergoing coronary angiography or intervention.. Circulation.

[12] (2012). KDIGO clinical practice guideline for acute kidney injury.. Kidney Int.

[13] Johannes T, Ince C, Klingel K, Unertl KE, Mik EG (2009). Iloprost preserves renal oxygenation and restores kidney function in endotoxemia-related acute renal failure in the rat.. Crit Care Med.

[14] Nasrallah R, Nusing RM, Hebert RL (2002). Localization of IP in rabbit kidney and functional role of the PGI(2)/IP system in cortical collecting duct.. Am J Physiol Renal Physiol.

[15] Rozhkov V, Wilson D, Vinogradov S (2002). Phosphorescent Pd porphyrindendrimers: Tuning core accessibility by varying the hydrophobicity of the dendritic matrix.. Macromolecules.

[16] Turker RK, Demirel E, Ercan ZS (1988). Iloprost preserves kidney function against anoxia.. Prostaglandins Leukot Essent Fatty Acids.

[17] Mizutani A, Okajima K, Uchiba M (2003). Antithrombin reduces ischemia/ reperfusion induced renal injury in rats by inhibiting leukocyte activation through promotion of prostacyclin production.. Blood.

[18] Isobe H, Okajima K, Uchiba M, Harada N, Okabe H (2002). Antithrombin prevents endotoxin-induced hypotension by inhibiting the induction of nitric oxide synthase in rats.. Blood.

[19] Johannes T, Mik EG, Nohe B, Raat NJ, Unertl KE, Ince C (2006). Influence of fluid resuscitation on renal microvascular PO2 in a normotensive rat model of endotoxemia.. Crit Care.

[20] Kohli HS, Bhaskaran MC, Muthukumar T (2000). Treatment-related acute renal failure in the elderly: a hospital-based prospective study.. Nephrol Dial Transplant.

[21] Santacruz F, Barreto S, Mayor MM, Cabrera W, Breuer N (1996). Mortality in elderly patients with acute renal failure.. Ren Fail.

[22] Pascual J, Liano F (1998). Acute Renal Failure Study Group. Causes and prognosis of acute renal failure in the very old.. J Am Geriatr Soc.

[23] Liano F, Pascual J (1996). Epidemiology of acute renal failure: a prospective, multicenter, community based study. Kidney Int.

[24] Fonarow GC, Heywood JT (2006). The confounding issue of comorbid renal insufficiency.. Am J Med.

[25] Zanchetti A, Stella A (1999). Cardiovascular disease and the kidney: an epidemiologic overview.. J Cardiovasc Pharmacol.

[26] Wencker D (2007). Acute cardio-renal syndrome: progression from congestive heart failure to congestive kidney failure.. Curr Heart Fail Rep.

[27] Liu YL, Prowle J, Licari E, Uchino S, Bellomo R (2009). Changes in blood pressure before the development of nosocomial acute kidney injury.. Nephrol Dial Transplant.

[28] Sutton TA, Fisher CJ, Molitoris BA (2002). Microvascular injury and dysfunction during ischemic acute renal failure.. Kidney Int.

[29] Imai E, Abe K (2013). Blood pressure drop in summer may cause acute kidney injury with irreversible reduction of glomerular filtration rate.. Clin Exp Nephrol.

[30] Hsu CY, Ordonez JD, Chertow GM, Fan D, McCulloch CE, Go AS (2008). The risk of acute renal failure in patients with chronic kidney disease.. Kidney Int.

[31] Benowitz NL (2003). Cigarette smoking and cardiovascular disease: pathophysiology and implications for treatment.. Prog Cardiovasc Dis.

[32] Heiss C, Amabile N, Lee AC (2008). Brief secondhand smoke exposure depresses endothelial progenitor cells activity and endothelial function: sustained vascular injury and blunted nitric oxide production.. J Am Coll Cardiol.

[33] Flouris AD, Vardavas CI, Metsios GS, Tsatsakis AM, Koutedakis Y (2009). Biological evidence for the acute health effects of secondhand smoke exposure.. Am J Physiol.

[34] Alonso-Coello P, Bellmunt S, McGorrian C (2012). American College of Chest Physicians: antithrombotic therapy in peripheral artery disease: antithrombotic therapy and prevention of thrombosis, 9th edn: American College of Chest Physicians evidence-based clinical practice guidelines.. Chest.

[35] Wu VC, Huang TM, Lai CF (2011). Acute-on-chronic kidney injury at hospital discharge is associated with long-term dialysis and mortality.. Kidney Int.

